# Perioperative methadone compared to placebo in elderly hip fracture patients: a study protocol for a randomized controlled trial (MetaHip trial)

**DOI:** 10.1186/s13063-024-08694-6

**Published:** 2024-12-20

**Authors:** Kevin Heebøll Nygaard, Thomas Strøm, Kirsten Specht, Sofie Ronja Petersen, Jesper Ougaard Schønnemann

**Affiliations:** 1 Department of Orthopedics, University Hospital of Southern Denmark, Kresten Philipsensvej 15, Aabenraa, 6200 Denmark; 2 Department of Anesthesiology and Intensive Care, University Hospital of Southern Denmark, Kresten Philipsensvej 15, Aabenraa, 6200 Denmark; 3 Center for COPD, Center for Health and Rehabilitation, Randersgade 60, Copenhagen Ø, 2100 Denmark; 4 Department of Clinical Research, University Hospital of Southern Denmark, Kresten Philipsensvej 15, Aabenraa, 6200 Denmark

**Keywords:** Perioperative methadone, Elderly, Frail, Hip fracture, RCT, Postoperative, Pain, Opioid consumption

## Abstract

**Background:**

Hip fractures are a source of severe pain among the elderly population and pose challenges due to limited analgesic tolerance. Perioperative methadone has shown promise in our pilot study suggesting a safe dose of 0.10 mg/kg, prompting further investigation into its benefits for elderly hip fracture patients.

**Methods:**

This study employs a double-blinded randomized controlled trial to assess the analgesic effects of a single dose of methadone during hip fracture surgery. Patients aged ≥ 60 years are consecutively enrolled and randomized to receive either perioperative methadone (treatment group) or a saline solution (placebo group). A sample size of 130 patients is required for 88% statistical power. The medication is administered intravenously at anesthesia induction and monitored until discharge. A follow-up observation is conducted 3 months post-surgery.

**Discussion:**

Primary outcome: Daily consumption of opioids within the first 3 days after surgery. Secondary outcomes include pain, mobility, nausea, vomiting, time to discharge, need for antidote, delirium, and constipation. The 3-month follow-up includes opioid use, pain, EQ-5D-5L scores, mobility, and persistent side effects. If statistically significant advantages are found in the treatment group, perioperative methadone could be considered as standard care for hip fracture patients, potentially enhancing their pain management. The study’s outcomes will provide insights into the feasibility and effectiveness of incorporating methadone into routine clinical practices for this patient group.

**Trial registration:**

ClinicalTrials.gov ID: NCT06086171, submitted 4. October 2023. EU-CT: 2023–506252-24–00, UTN: U1111-1294–6125.

**Supplementary Information:**

The online version contains supplementary material available at 10.1186/s13063-024-08694-6.

## Administrative information

Note: the numbers in curly brackets in this protocol refer to SPIRIT checklist item numbers. The order of the items has been modified to group similar items (see http://www.equator-network.org/reporting-guidelines/spirit-2013-statement-defining-standard-protocol-items-for-clinical-trials/).

**Table:** 

Title {1}	Perioperative methadone compared to placebo in elderly hip fracture patients: a study protocol for a randomized controlled trial (MetaHip trial).
Trial registration {2a and 2b}.	ClinicalTrials.gov ID: NCT06086171 EU-CT: 2023–506252-24–00 UTN: U1111-1294–6125
Protocol version {3}	03.12–24 ver. 2
Funding {4}	The study is non-commercial. Running costs: External grants from The A.P. Møller and Chastine Mc-Kinney Møller Foundation (50.000 kr.) and Knud and Edith Eriksens memorial foundation (40.000 kr.) Scholarships: The Region of Southern Denmark (1. Year) and University Hospital of Southern Denmark (2. + 3. Year) The financing does not affect study completion or results.
Author details {5a}	^1^: Department of orthopedics, University hospital of southern Denmark, Kresten Philipsensvej 15, 6200 Aabenraa ^2^: Department of anesthesiology and intensive care, University hospital of southern Denmark, Kresten Philipsensvej 15, 6200 Aabenraa ^3^: Center for COPD, Center for Health and Rehabilitation, Randersgade 60, 2100 København Ø ^4^: Department of clinical research, University hospital of southern Denmark, Kresten Philipsensvej 15, 6200 Aabenraa †: Corresponding author
Name and contact information for the trial sponsor {5b}	Jesper Ougaard Schønnemann E-mail: Jesper.Ougaard.Schoennemann1@rsyd.dk Phone: + 4,579,976,170
Role of sponsor {5c}	The trial sponsor is also the principal investigator and head of the research group. He contributed substantially to the trial design and will contribute to the collection and interpretation of data, as well as writing of the report. We will submit the report for publication undeterred by the study results. We make decisions democratically within the research group. Thus, study sponsor does not have ultimate authority.

## Introduction

### Background and rationale {6a}

Hip fractures are recognized as the most serious consequence of osteoporosis and low-energy trauma [1]. With the expanding elderly population, the global incidence of hip fractures is expected to rise from 1.66 million in 1990 to 6.26 million by 2050 rendering them an established health problem worldwide [1].

Hip fractures are associated with severe pain and thus require adequate analgesic treatment [2, 3]. However, elderly individuals, who often have a reduced tolerance for analgesic medications [4, 5], are the primary population affected by hip fractures. The demand for adequate pain relief combined with a low tolerance for analgesic drugs makes the analgesic treatment of elderly hip fracture patients difficult and calls for further studies on the subject. Furthermore, studies have shown that elderly patients who take up opioid treatment often develop a chronic use [6–8].

Studies investigating the perioperative use of methadone have shown promising analgesic properties [9–13]. Globally, perioperative methadone has been investigated for its potential to improve pain management while reducing postoperative opioid consumption. In the USA and Canada, studies and systematic reviews have demonstrated methadone’s efficacy in lowering opioid use and improving pain control across various surgical populations [14, 15]. However, research in Europe remains limited, with methadone’s role in perioperative care underexplored, particularly in older patients [16]. This trial seeks to address this gap by evaluating methadone in a previously unstudied demographic, contributing to the broader understanding of its application in orthopedic procedures and older individuals.

Our pilot study [17] showed that a dosage of 0.10 mg/kg was the maximal dose for this patient group and showed promising analgesic properties. With the present study, we will further investigate the advantages of methadone in elderly hip fracture patients by conducting a randomized controlled trial. The trial will include patients ≥ 60 years old who are admitted to a regional hospital with an acute hip fracture.

Sufficient management of acute postoperative pain is important concerning morbidity, hospital costs, and mortality [11, 12]. Sufficient analgesic treatment is crucial in the initial postoperative days, which are considered the most painful phase of recovery [11]. Opioids have conventionally been used as an analgesic treatment in this phase. However, this treatment has been accompanied by side effects and addiction [13]. The most common opioid-related side effects include constipation, nausea, itchy skin, dry mouth, vertigo, and sedation [6, 13]. Methadone shares these side effects; however, seeing that methadone only needs to be administered one time, the risk of side effects decreases significantly.

### Objectives {7}

We hypothesize that the utilization of a single dose of perioperative methadone may lead to a reduction in pain severity, a decrease in morphine consumption, mitigation of postoperative complications, an enhancement in analgesic effectiveness, an acceleration of recovery, and facilitation of early hospital discharge following hip fracture surgery.

To investigate this hypothesis we established the following trial objectives:

1. To investigate the analgesic effect of a single dose of perioperative methadone compared with a placebo in acute hip fracture surgery.
2. To investigate the long-term effects of this methadone dose on continued opioid consumption, pain, and mobility 3 months after surgery.

### Trial design {8}

The design of the study is a double-blinded randomized controlled trial (RCT) with two arms as parallel groups investigating the effects of perioperative methadone (treatment group) compared with a saline solution (placebo group). We test the superiority of methadone as compared to our standard analgesic regime containing peripheral nerve block, morphine, and paracetamol.

## Methods: participants, interventions, and outcomes

### Study setting {9}

The study setting is a Danish regional hospital in the emergency- and orthopedic departments. The study will consecutively include patients presenting with an acute hip fracture on x-rays. Data collection takes place in the orthopedic ward. After discharge, the patients will receive a phone call from the investigator as a follow-up 3 months after surgery. The full details regarding the trial site are available at Clinical Trials Information System (CTIS): https://euclinicaltrials.eu/search-for-clinical-trials/?lang=en&EUCT=2023-506252-24-00.

### Eligibility criteria {10}

#### Inclusion criteria

1. Patients diagnosed with an acute hip fracture (incurred < 24 h ago) on x-rays in the emergency department (ED) at the University Hospital of Southern Denmark (UHS). This includes collum femoris fractures, pertrochanteric fractures, and subtrochanteric fractures.
2. Age ≥ 60 years.
3. Patients must be able to read and understand Danish.

#### Exclusion criteria

Health conditions preventing treatment:

1. Multiple fractures or multi-trauma patients.
2. Previous allergic reactions or hypersensitivity towards methadone hydrochloride or sodium chloride.
3. Chronic obstructive pulmonary disease with either past exacerbations or daily symptoms (Gold classification C + D).
4. History of acute asthma attacks.
5. History of drug-induced eczema.
6. Pulmonary hypertension.
7. Raised intracranial pressure or recent head injury.
8. Pheochromocytoma.
9. History of paralytic ileus.
10. QT-interval prolongation on electrocardiogram (ECG) (> 500 ms).
11. Myasthenia gravis.
12. Known liver disorder.
13. Hypotension (systolic blood pressure < 100 mmHg at admission).
14. Acute pancreatitis.
15. Severe kidney disease (GFR ≤ 10).

Other exclusion criteria:

1. Concurrent administration with MAO inhibitors or within 2 weeks of suspending treatment with these medicinal products.
2. Concurrent administration of benzodiazepines.
3. Impaired cognitive function, e.g., dementia. (Patients must be able to give informed consent and be able to ask for rescue analgesics if needed).
4. Current opioid addiction or intravenous addiction.

Notes:

Although methadone is considered safe in patients with renal dysfunction or chronic renal disease [18, 19], the SmPC lists severe kidney disease as a contraindication. As a result, we will exclude patients with a Glomerular Filtration Rate (GFR) of ≤ 10 from the study.

We do not test female patients for pregnancy, as they are ≥ 60 years.

### Who will take informed consent? {26a}

Patients with an acute hip fracture will be included at the ED. The orthopedic physician will screen every hip fracture patient ≥ 60 years old for inclusion and will inform eligible patients about the study. We will provide verbal and written information to patients, allowing them 2 h to consider. The orthopedic physician will greet and examine the patient in the ED within 4 h from arrival as per hospital standards. During this interaction, the orthopedic physician will provide the written information about the study and present the verbal information in an easy-to-understand language without technicalities or value-laden phrases. The orthopedic physician presenting the information will ensure the patient understands it well, potentially by asking questions or having the patient repeat key details. Subsequently, the orthopedic physician will leave the patient for a minimum of 2 h to allow them to consider. The physician will address any questions or ambiguities upon their return, ensuring that consent is informed and voluntary before obtaining it.

### Additional consent provisions for collection and use of participant data and biological specimens {26b}

Participants provide written consent for participation and have the option to withdraw it at any time. Any person sensitive data will be processed in the study according to the General Data Protection Regulation, the Danish Data Protection Act, and the Danish Health Act. Any information kept on paper will be stored in a locked cabin within a locked office. Any electronic data is stored in REDCap (Research Electronic Data Capture, hosted at Odense University Hospital), where access is limited to specified users. We do not collect any biological specimens.

## Interventions

### Explanation for the choice of comparators {6b}

As no identical placebo is available, we use a standard saline solution as placebo prepared by un-blinded personnel. Using consecutive randomization numbers and custom labeling on the medicine packages the Registered Nurse (RN) is able to prepare the medicine according to the treatment allocation.

### Intervention description {11a}

The treatment group receives methadone hydrochloride, while the placebo group receives a standard saline solution. A certified registered nurse (RN) anesthetist administers the investigational medicine intravenously at the induction of anesthesia (no later than 10 min before knife-to-skin). The patients only receive this one dose. We will use a dosage of 0.10 mg/kg, as our previous study [17] confirmed its safety for elderly hip fracture patients.

The orthopedic doctor responsible for inclusion will randomize patients using the randomization module in REDCap. A block randomization method with selected block sizes of four and six, stratified by gender and type of anesthesia (general or spinal anesthesia), generates an irreversible, random allocation sequence. We assign patients in a 1:1 ratio to either the treatment or placebo group. Each included patient receives a random treatment number corresponding to an ampoule containing either methadone or placebo. Glostrup Pharmacy retains an un-blinded list of treatment numbers and their respective treatment groups for emergencies. RNs in the ED prepare the investigational medicine and place it near the patient awaiting surgery. The un-blinded RN is not involved in patient treatment once the patient leaves the ED.

### Criteria for discontinuing or modifying allocated interventions {11b}

Criteria for discontinuing allocated intervention:

- If a patient no longer wishes to participate
- If, for some reason, investigational medicine cannot be administered
- If the patient or personnel has failed to follow project protocol
- If any severe or life-threatening adverse reaction (SAR or SUSAR) were to occur
- If an error has been made during the evaluation of inclusion- or exclusion criteria

### Strategies to improve adherence to interventions {11c}

Given that we administer the investigational medicine only once, compliance with the allocated intervention is expected. A certified RN anesthetist administers the medicine using a blinded syringe prepared by an un-blinded RN from the ED. This process ensures that the personnel involved in patient treatment remain blinded.

### Relevant concomitant care permitted or prohibited during the trial {11d}

We allow concurrent analgesic treatment following hospital guidelines. This encompasses the utilization of peripheral nerve blocks before surgery and subsequent analgesic management after surgery. The primary analgesic approach comprises rescue morphine and paracetamol; however, orthopedic physicians can customize the analgesic approach to ensure adequate pain control, e.g. by supplementing with long-acting opioids. We will convert the dosage of any prescribed opioids after surgery into morphine equivalents and record it in our dataset. Utilizing epidurals in the treatment of hip fracture patients is counterproductive as they impede mobilization, the cornerstone of their rehabilitation. Thus, epidurals are not routinely used for these patients.

### Provisions for post-trial care {30}

Three months after surgery patients will receive a phone call from the investigator and will jointly complete the final chart. Patients will not receive any reimbursement for participating. Study participants are covered by the usual patient insurance owned by UHS and the Danish Patient Compensation.

### Outcomes {12}

#### Primary outcome

- *Daily consumption of opioids*: This includes both short-acting and long-acting opioids and we calculate a daily dose for the first 3 days after surgery. We convert different types of opioids into the daily morphine equivalent dose.

#### Secondary outcomes

- *Postoperative pain assessment:* We ask patients to assess pain intensity in the hip both at rest and when mobilized. We evaluate pain intensity using the verbal rating scale (VRS) since this method is valid for hip fracture patients [20]. The patients will assess pain intensity once daily for the first three postoperative days.
- *Standing up*: We register the number of hours before standing up for the first time postoperatively.
- *Cumulated Ambulation Score (CAS)*: Physiotherapists at the ward routinely use the CAS to assess the mobility of patients. We record the score once daily for the first three postoperative days.
- *Postoperative nausea or vomiting (PONV)*: We register PONV binomial as “yes” or “no” once daily for the first three postoperative days.
- *Time to discharge*: We record the number of days from the arrival at the ward until a patient is ready for discharge. The orthopedic doctor doing ward rounds decides when a patient is ready for discharge. Patients moved to a different ward will still be considered hospitalized and in this case, it is registered that the patient needs further hospitalization and the collection of further data stops.
- *Need for antidote*: We document the administration of antidote during admission binomial as “yes” or “no” once daily for the first three postoperative days. Indications for the use of an antidote include a respiratory frequency of < 10/min with peripheral oxygenation of < 94% despite 4 L of oxygen/minute, clinical signs of opioid overdose e.g. disproportionate drowsiness, or if the orthopedic resident deems it necessary.
- *Confusion Assessment Method (CAM)*: We monitor patients using CAM for signs of delirium the first 3 days after surgery. The method is binomial.
- *Constipation***:** We document the occurrence of constipation during admission binomial as “yes” or “no”. We consider a patient with no bowel movements for ≥ 2 days as constipated. This will be registered once daily for the first three postoperative days.

#### Three-month follow-up

- *Continued opioid consumption*: We report opioid consumption as a mean daily consumption of both long-acting opioids and rescue medication prescribed in the medication chart and reported by the patient. We convert different types of opioids into the daily morphine equivalent dose.
- *Pain*: We ask patients to assess their level of pain using the VRS at rest and when mobilized.
- *EQ-5D-5L*: The investigator will fill out this validated generic questionnaire with the patient, which has been used for hip fracture patients in previous studies [21].
- *New Mobility Score (NMS)*: We assess patient mobility using the NMS as seen in other studies [22].
- *Persistent side effects*: We document potential persistent adverse reactions, such as vertigo, nausea, vomiting, constipation, and significant or debilitating drowsiness or dizziness. We file these observations systematically in the follow-up chart.

### Participant timeline {13}


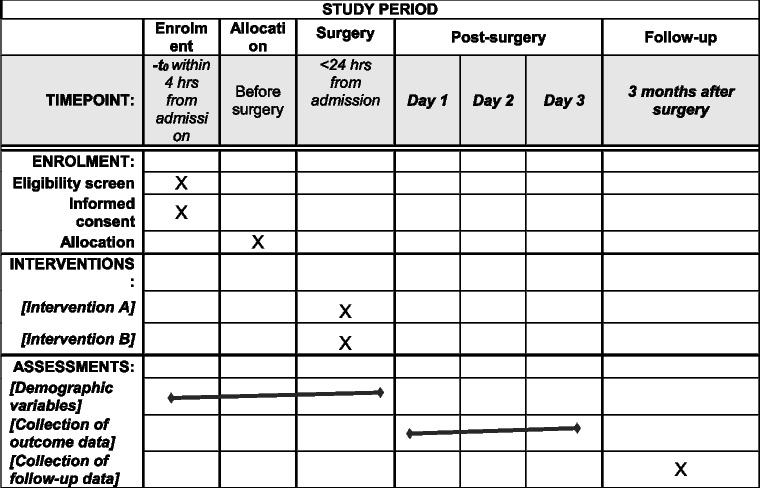


### Sample size {14}

To determine the optimal sample size for this RCT, we performed a power calculation for a Poisson regression with clustered standard errors. We assumed that our primary outcome (morphine consumption) was Poisson distributed. Hence, the mean in the two groups should be equal to the variance. Consequently, if the variance was larger than the mean, we would expect the excess variation to be due to intra-personal variance. Looking through the literature, we found that cognitively intact patients who sustain a hip fracture have a mean daily dose of parenteral morphine sulfate equivalents of 2.6 mg with a standard deviation (SD) of 4.55 [3]; therefore, the intra-personal variance was set to 18. A clinically significant decrease in the consumption of parenteral morphine sulfate was set to 33.33%. With a significance level of 0.05, we deemed 65 patients in each treatment arm (130 in total) sufficient to achieve a power of 88%.

### Recruitment {15}

From other studies investigating hip fracture patients, such as the SENSE trial [22] and our pilot study [17], we experience that roughly 30% of eligible patients are enrolled in the study. This means we need 390 eligible patients to include the 130 patients needed in total. At UHS approximately 30 hip fracture patients are treated each month. Thus, our inclusion period would require being 12–13 months as a minimum. We terminate the study when we have included 130 patients in total.

## Assignment of interventions: allocation

### Sequence generation {16a}

An irreversible, random allocation sequence will be generated using block randomization with selected block sizes of 4 and 6, which will be stratified on gender and type of anesthesia (general anesthesia or spinal anesthesia). We assign patients to the trial with an allocation of 1:1 to either treatment or placebo. Included patients receive a random treatment number that corresponds to one pre-packaged ampoule. The ampoules are stored in blinded boxes where treatment and placebo are mixed. We keep an un-blinded list of treatment numbers and corresponding treatment group at Glostrup Pharmacy for emergencies.

### Concealment mechanism {16b}

Un-blinded RNs from the ED prepare the blinded investigational medicine according to the treatment number. Prepared medicine consists of a syringe with clear content and cannot be distinguished. The RN preparing the syringe is no longer involved in the treatment once the patient has left the ED. The patient and all other personnel involved remain blinded.

### Implementation {16c}

The included orthopedic doctor will randomize patients using the randomization module in REDCap. This generates an individual treatment number that corresponds to the treatment group.

## Assignment of interventions: blinding

### Who will be blinded {17a}

We ensure the blinding of the patient and involved personnel including the study investigator, the orthopedic doctor, the RN anesthetist, and the RNs at the ward. We achieve blinding by using individual treatment numbers that correspond to a custom label on the medicine ampoule. The only un-blinded personnel are the RNs from the ED who prepare the syringe. The content in the prepared syringe cannot be distinguished.

### Procedure for unblinding if needed {17b}

Un-blinding will only occur when the statistical analysis is complete or in case of an emergency, in which case Glostrup Pharmacy offers a 24-h hotline service with knowledge of the respective treatment groups. The principal investigator is responsible for the un-blinding in case of emergencies.

## Data collection and management

### Plans for assessment and collection of outcomes {18a}

Data will be collected by trained registered nurses (RNs) from the ward to ensure accuracy and completeness. Any missing data is expected to occur randomly due to the training and standardized procedures in place. Statistical analysis will be performed only on data from patients who have received the investigational medication; data from patients who did not receive the investigational medication will be excluded. The primary and secondary outcomes (detailed below) will be recorded on standardized charts. These charts will be entered into our REDCap database. Three months after surgery, patients will be contacted by the investigator for a follow-up phone call to complete the final chart and the EQ-5D-5L questionnaire.

#### Primary outcome

##### Daily consumption of opioids

Postoperative opioid consumption will be measured as the mean daily consumption of rescue and long-acting morphine equivalents within the first 72 h after surgery. The morphine equivalent dose for various opioids will be calculated. The source of this data is the medical chart, with RNs recording the amounts on the observation chart, which is subsequently entered into REDCap.

#### Secondary outcomes

##### Postoperative pain assessment

Patients will assess pain intensity in the hip at rest and during mobilization using the Verbal Rating Scale (VRS). Pain intensity will be assessed once daily for the first three postoperative days. This data will be sourced from the observation chart.

##### Time to standing up

The number of hours until the patient first stands postoperatively will be recorded. This data will be sourced from the electronic patient record, which logs timestamps such as time from knife-to-skin and return to the ward.

##### Cumulated Ambulation Score (CAS)

Physiotherapists routinely use the CAS to assess patient mobility. This score will be recorded once daily for the first three postoperative days on the observation chart.

##### Postoperative Nausea or Vomiting (PONV)

The occurrence of PONV will be recorded as “yes” or “no” once daily for the first three postoperative days on the observation chart by the RNs.

##### Time to discharge

The number of days from arrival at the ward until the patient is deemed ready for discharge will be recorded. This decision is made by the orthopedic doctor during ward rounds. If a patient is transferred to another department, they are still considered hospitalized, and data collection stops. This data will be sourced from timestamps in the electronic patient record and entered into REDCap.

##### Need for an antidote

The administration of an antidote during admission will be recorded as “yes” or “no” once daily for the first three postoperative days on the observation chart. The source of this data is the medical chart.

##### Confusion Assessment Method (CAM)

Patients will be monitored for signs of delirium using the CAM for the first 3 days after surgery. The method records 0 for no signs of delirium and 1 for delirium. Data will be sourced from the electronic patient record and recorded on the observation chart.

##### Constipation

The occurrence of constipation during admission will be recorded on the observation chart by the RNs.

#### Three-month follow-up

##### Continued opioid consumption

Continued opioid consumption will be recorded directly into REDCap as the mean daily consumption of both long-acting opioids and rescue medication, based on the medication chart and patient report. Various opioids will be converted into daily morphine milligram equivalents.

##### Pain

Patients will assess their pain level using the VRS at rest and during mobilization on the follow-up day. Data will be entered directly into REDCap.

##### EQ-5D-5L

The investigator will complete this validated generic questionnaire with the patient. The EQ-5D-5L, previously used in studies with hip fracture patients [21], can be completed directly in REDCap using the patient’s responses.

##### Mobility

Patients’ mobility will be assessed using the New Mobility Score, as utilized in other studies [22]. The score will be calculated directly in REDCap based on patient statements.

##### Persistent side-effects

The presence of persistent adverse reactions, including dizziness, vertigo, nausea, vomiting, constipation, and drowsiness, will be recorded based on patient statements and entered directly into REDCap

### Plans to promote participant retention and complete follow-up {18b}

RNs at the ward manage data collection. This way the participant does not have any trial-specific activities and can focus on their rehabilitation. Furthermore, we administer the study drug in the context of surgery and only this one time. Consequently, we expect full participant retention as participation involves no inconvenience.

Seeing that the expected mean age of the participants is 80–82 years old, we refrain from any electronic follow-up questionnaires. Instead, we rely on a phone call, which should be familiar to the participants. This is also a lot less inconvenient than a follow-up visit at the hospital. We collect no data from participants who discontinue intervention protocols. However, we register any serious adverse reaction for 12 days after study drug administration for all patients. This is in accordance with Good Clinical Practice (GCP) where serious adverse reactions need to be registered for five half-lives.

### Data management {19}

The principal investigator will conduct a list of all patients screened for inclusion. The list will cover both included and excluded patients and include patient number, CPR number, and initials. Furthermore, an identification list will be conveyed by the principal investigator listing all included patients with patients’ full name, CPR number, and patient study number. Both lists will be stored in a locked office within a locked cabin. The principal investigator ensures direct access to any data, documents, or journals in case of monitoring, audits, or inspections from the GCP units and The Danish Medicine Agency. We use double data entry where trained RNs fill out a standardized observation chart. The study investigator later enters these data into our secure REDCap database and double checks data values.

For every included patient, an eCRF will be completed. Any editing will be logged so that original data can be retrieved at any time. The log will also show the time any data has been edited and by whom. Demographic data regarding each patient is retrieved from the journal and registered in the eCRF. This includes age, gender, co-morbidities, diagnosis (fracture type), height, weight, medicine consumption, drug addiction, smoking status, and the type of anesthesia used.

### Confidentiality {27}

Any person-sensitive data processed in the study is managed according to the General Data Protection Regulation, the Danish Data Protection Act, and the Danish Health Act. Any information kept on paper will be stored in a locked cabin within a locked office. Any electronic data is stored in REDCap, where access is limited to specified users.

### Plans for collection, laboratory evaluation, and storage of biological specimens for genetic or molecular analysis in this trial/future use {33}

We do not use any biological specimens in this trial.

## Statistical methods

### Statistical methods for primary and secondary outcomes {20a}

#### Primary outcome

To investigate if the mean consumption of opioids differs between groups, a mixed-effects Poisson model will be utilized. To assess the model’s fit, normality of the deviance residuals will be graphically checked for each time point. If the model does not provide a good fit, the mean structure will be further investigated, and/or a mixed-effects negative binomial model will be conducted. Seeing that our RNs are trained in collecting data and the fact that we use standardized collection charts it is safe to assume that any missing data will be missing at random. The maximum likelihood method in the mixed-effects Poisson model accounts for the missingness and gives valid results in case the data are missing at random. Thus, no action will be undertaken on missing data.

#### Secondary outcomes

For categorical variables (e.g., nausea and vomiting, need for an antidote, and delirium), Fisher’s exact test or the chi-square test will be used to determine if there is a significant difference in distribution between the two groups.

For continuous variables (e.g., VRS, CAS, and time to discharge), Poisson or negative binomial regression will be utilized to assess if the distributions between the two groups are similar. In the case of longitudinal data for the above-mentioned variables (i.e., opioid consumption, VRS, time to discharge, and CAS), the *p*-value will be based on the contrast over all time points (if the treatment-time interaction is included as a separate term in the model) to account for multiplicity. To further address multiplicity and reduce the risk of type 1 errors, adjustments for multiple testing may be applied in the analysis of secondary outcomes.

Any deviations from the statistical plan mentioned above will be transparently reported and justified in our final publication. If we suspect any data to be falsified or incorrect, it will be disregarded.

### Interim analyses {21b}

We do not plan to conduct any interim analyses. There are no stopping guidelines. However, the study will be terminated ahead of time if we suspect that the investigational medicine is affecting patient safety or is negatively affecting the course of admission.

### Methods for additional analyses (e.g., subgroup analyses) {20b}

We stratify participants on the type of anesthesia and gender. Thus, separate analyses for general anesthesia and spinal anesthesia can be explored. Additional subgroup analyses may be explored if deemed relevant to further understand the outcomes. However, no other adjusted analyses are currently planned.

### Methods in analysis to handle protocol non-adherence and any statistical methods to handle missing data {20c}

Seeing that our RNs are trained in collecting data and the fact that we use standardized collection charts it is safe to assume that any missing data will be missing at random. The maximum likelihood method in the mixed-effects Poisson model accounts for the missingness and gives valid results in case the data are missing at random. In case of missing data in our multiple testing modalities, the p-value will be based on the contrast over all time points (if the treatment-time interaction is included as a separate term in the model) which accommodates for multiplicity. Thus, no action will be undertaken on missing data. Patients who do not adhere to the protocol will be disregarded.

### Plans to give access to the full protocol, participant-level data and statistical code {31c}

This paper acts as public access to the full study protocol. Anonymized participant-level data and statistical code can be provided upon request.

## Oversight and monitoring

### Composition of the coordinating center and trial steering committee {5d}

We have established a trial steering committee to provide rigorous oversight throughout the trial process. The steering committee will convene monthly during the initial phases and subsequently every 2–3 months to review progress and address any emerging issues. Additionally, the committee is responsible for overseeing day-to-day trial operations, ensuring adherence to protocol guidelines and ethical standards. The steering committee retains the flexibility to convene more frequently or on short notice as needed, allowing for timely responses to unforeseen circumstances or urgent matters. This proactive approach ensures the trial’s integrity and facilitates effective decision-making.

### Composition of the data monitoring committee, its role and reporting structure {21a}

We use external monitoring from the unit of Good Clinical Practice (GCP unit) at OPEN for Odense University Hospital. This unit is independent from the sponsor and any competing interests. Further details are found on their website: https://gcp-enhed.dk/english/

The national units for good clinical practice (GCP units) will monitor the study throughout its course from initiation to termination. Any data from the study will be stored securely on SharePoint or in REDCap by Danish law. Thus, fulfilling data storage standards. The principal investigator and delegated co-investigator construct a Trial Master File (TMF), which will be edited continuously. The TMF exists as a hybrid with some content stored electronically on SharePoint while live documents are stored on paper in a locked cabin within a locked office.

### Adverse event reporting and harms {22}

Any adverse event is registered by the investigator in the electronic case report form (eCRF) with documentation of severity, time of the event, assessment of causation with investigational medicine, and evaluation of the event being expected or unexpected. Any adverse event, besides serious adverse events, will be reported to the trial sponsor upon study completion.

If the study extends for more than 1 year an annual safety report will be uploaded in CTIS. Any serious adverse event or protocol deviation is registered by the investigator in the electronic case report form within 24 h and REDCap will automatically report to the trial sponsor via email. The trial sponsor is responsible for the registration of any lethal or life-threatening Suspected Unexpected Serious Adverse Reaction (SUSAR) and is obligated to file a report in EudraVigilance within 7 days. The trial sponsor is accountable that information regarding any SUSAR, which is not lethal or life-threatening, is registered and reported to The Danish Medicine Agency within 15 days in EudraVigilance.

The trial sponsor is responsible for continuous assessment of the risk/benefit ratio regarding the study. Any circumstances affecting patient safety or study conduction should be reported to The Danish Medicine Agency immediately. These circumstances should also be reported to any involved physicians and the National Committee on Health Research Ethics.

The sponsor should announce all relevant information regarding the sponsor and investigator’s follow-up on the incident. This should be announced within 8 days after reporting the incident. Any report is accompanied by comments on the eventual consequences of the study. The product resume (SmPC) from Streuli will be used as a reference when evaluating if any event is expected or unexpected.

In case of an emergency, e.g., serious adverse event (SAE), the principal investigator is responsible for making necessary procedures and expertise available. In the case of advert events (AE), the patient will be monitored until satisfactory recovery or stabilization has been reached. Any ongoing AE or SAE when a patient is being discharged will be treated and monitored by the general practitioner who will be notified in the epicrisis.

#### Events that will not be reported

AE which are common and harmless following hip fracture surgery or anesthesia will not be reported. These include:

- Drop in hemoglobin until 4.5 mmol/L
- Vertigo/dizziness
- Decrease in blood pressure until systolic pressure of 100 mmHg
- Rise in leukocytes and CRP
- Fever until 38.5 ℃
- Confusion or delirium
- Nausea or loss of appetite
- Vomiting
- Stomach pain
- Constipation
- Sleepiness/drowsiness
- Increased heart rate until 100 beats pr. minute
- Headache

Some SAEs following hip fractures require prolonged admission or supplementary treatment but have no relation to the investigational medicine and thus will not be reported. These include:

- Pneumonia
- Urinary infection
- Infection with no known focus
- Delirium
- Hypertensive lung edema
- Dehydration

### Frequency and plans for auditing trial conduct {23}

#### Coordinating center and organizational structure

The orthopedic department serves as the coordinating center for this trial. It is responsible for the day-to-day operational and logistical aspects, including maintaining communication between the trial groups and ensuring protocol adherence.

#### Trial oversight committees

*Trial steering committee (TSC)*: The TSC includes the trial sponsor and supervisory team, tasked with overseeing trial conduct, safety, and progress. The committee will meet monthly during the initial phases and subsequently every 2–3 months.

*Project management group (PMG):* The PMG consists of the sponsor, three delegated co-investigators, and attending physicians at the orthopedic department. This group manages any day-to-day operational aspects. Weekly meetings will ensure efficient trial conduct and address any operational challenges.

#### Auditing plan

Independent auditing of trial conduct will be performed by the national Good Clinical Practice (GCP) unit. It is expected that audits will occur approximately every 25–30 enrolled patients, corresponding to intervals of approximately 3 months. Audits will focus on verifying compliance with protocol requirements and regulatory standards.

#### Ethics and risk assessment

A Data Monitoring and Ethics Committee (DMEC) is not planned for this trial, as the intervention is classified as low risk based on the following considerations:

- Methadone’s established safety profile in similar contexts under controlled use.
- The administration of a relatively small, one-time-only dose.
- Close monitoring of all patients during the perioperative period, with predefined protocols for managing adverse events.
- Regular independent audits conducted by the GCP unit.

These measures ensure robust oversight while aligning with the trial’s low-risk designation.

### Plans for communicating important protocol amendments to relevant parties (e.g., trial participants, ethical committees) {25}

The trial sponsor and principal investigator are committed to ensuring that all personnel involved in the study possess the requisite education, instructions, and information to conduct the research in adherence to the established protocol. This commitment extends to maintaining ongoing training and support to uphold protocol integrity throughout the study duration.

Any proposed amendments to the protocol are diligently evaluated, and if deemed necessary, are promptly submitted to the Clinical Trial Information System (CTIS) for review. Prior to implementation, amendments must receive approval from the appropriate regulatory and ethical authorities, safeguarding the integrity and ethical conduct of the trial.

### Dissemination plans {31a}

Analysis of study data is commenced when inclusion of all patients has been completed. The principal investigator will then compile a study report in collaboration with the trial sponsor and additional research group as soon as possible and no later than 1 year after the trial has ended. The report will be submitted in CTIS and will serve as a draft for future manuscripts. The final manuscript will be read and approved by every author and published in an international, peer-reviewed scientific journal regardless of results being either positive, negative or inconclusive. Subsequently, data will be published on clinicaltrialsregister.eu.

## Discussion

The design of the study is a double-blinded randomized controlled trial (RCT) with two arms as parallel groups investigating the effects of perioperative methadone (treatment group) compared with a saline solution (placebo group). Our previous study (EudraCT no.: 2022–001857-22) [17] revealed the maximal tolerable dose of 0.10 mg/kg for elderly hip fracture patients. The study also revealed promising analgesic properties of perioperative methadone. However, the next step in generating scientific evidence on the subject is to test the superiority of methadone as compared to our standard analgesic regime containing morphine. Consequently, further testing using a randomized controlled trial is necessary. Furthermore, methadone has limited testing in elderly patients undergoing acute surgery leaving the body of evidence scarce. This rules out other study types like meta-analyses and systematic reviews. Methadone has many advantages and would potentially revolutionize the analgesic treatment of elderly and fragile patients. Accordingly, establishing sufficient evidence on the subject is important. Our previous study was a prospective cohort study. In order to move up the hierarchy of scientific evidence we have to conduct a randomized controlled trial. Seeing that pain is very subjective the trial needs to be double-blinded to avoid any bias.

### Practical implications of the study

This study has several practical implications for patient care, healthcare professionals, and the broader healthcare system:

For patients: By exploring the safety and efficacy of perioperative methadone in older hip fracture patients, the study aims to improve pain management while potentially reducing the reliance on postoperative rescue opioids. This could lead to better recovery outcomes and fewer opioid-related complications, such as sedation and constipation.

For healthcare professionals: The findings may offer evidence to support the integration of methadone into multimodal analgesia protocols. This could enhance perioperative pain management strategies and inform clinical decision-making in similar patient populations.

For the healthcare system: By reducing opioid consumption and its associated adverse effects, the study could lead to more efficient use of healthcare resources. Improved pain control may reduce hospital stays, readmissions, and the need for interventions to manage opioid-related side effects.

For research: The results will add to the growing body of evidence on methadone’s perioperative role, providing a foundation for future trials and contributing to the development of international guidelines.

## Trial status

We are currently recruiting patients, May 2024.

Protocol version number and date: Version 5, dated 25th August 2023, published on CTIS.

Date recruitment began: 9th November 2023.

Approximate date when recruitment will be completed: end of 2024.

## Supplementary Information

Supplementary Material 1. SPIRIT checklist.

## Data Availability

The present study will be conducted according to this protocol and according to GCP guidelines regarding quality control and assurance (ICH-GCP guidelines). Furthermore, the study will be conducted according to the European clinical trials regulation (EU No 536/2014). The trial sponsor is accountable for the management and filing of data according to applicable laws. Rights regarding the data, which consists of eCRFs in REDCap, are reserved by the orthopedic department at UHS and will be securely stored electronically for 25 years by OPEN. The Principal investigator will have access to filed data throughout the study and storage period. The principal investigator is responsible for reporting any deviations from this protocol using REDCap, which will generate an e-mail to the trial sponsor. The present protocol conforms to the Standard Protocol Items: Recommendations for Interventional Trials (SPIRIT) [23]. The project will also be registered on clinicaltrials.gov before initiation.

## Abbreviations

RCT: Randomized controlled trial
ED: Emergency department
SHS: University Hospital of Southern Denmark
MAO inhibitors: Monoamine oxidase inhibitors
QT-interval: The Q-T segment on an electrocardiogram
ECG: Electrocardiogram
VRS: Verbal rating score
REDCap: Research Electronic Data Capture
PACU: Post-anesthesia care unit
PONV: Postoperative nausea or vomiting
CAS: Cumulated ambulation score
CAM: Confusion assessment method
RN: Registered nurse
eCRF: Electronic case report form
CTIS: Clinical Trials Information System
GCP: Good clinical practice
TMF: Trial Master File
SAE: Serious adverse event
SAR: Serious adverse reaction
SUSAR: Serious unexpected suspected adverse reaction
AE: Adverse event
SPC or SmPC: Summary of Product Characteristics
GFR: Glomerular filtration rate
